# The relationship between cognitive and reading skills in typically developing pre-primary school children

**DOI:** 10.3389/fnbeh.2026.1832008

**Published:** 2026-07-01

**Authors:** Özden Erkan Oğul, Fatma Aydın, Gülsüm Uzer, Kıvanç Kök, Derya Aydın, Sibel Deniz, Mustafa Otrar, Şeyda Cankaya, Lütfü Hanoğlu, Burak Yuluğ

**Affiliations:** 1 Department of Ergotherapy, Faculty of Health Sciences, Istanbul Medipol University, Istanbul, Türkiye; 2 Brain and Cognition Research Center (BEYKOG), İstanbul Medipol University, Istanbul, Türkiye; 3 Neuroscience PhD Program, Graduate School of Health Sciences, Istanbul Medipol University, Istanbul, Türkiye; 4 Department of Biostatistics and Medical Informatics, International School of Medicine, Istanbul Medipol University, Istanbul, Türkiye; 5 Fatih Sultan Mehmet Primary School, Ministry of National Education, Istanbul, Türkiye; 6 Directorate General of Special Education and Guidance Services, Istanbul, Türkiye; 7 Department of Neurology, Faculty of Medicine, Alanya Alaaddin Keykubat University, Antalya, Türkiye; 8 Department of Neurology, School of Medicine, Istanbul Medipol University, Istanbul, Türkiye; 9 Department of Neurology, Faculty of Medicine, Alanya Alaaddin Keykubat University, Antalya, Türkiye

**Keywords:** children, executive function, reading comprehension, visual perception, working memory

## Abstract

**Aim:**

The aim of this study was to investigate the prospective relationship between cognitive abilities assessed at the beginning of the academic year and reading skills acquired by the end of the academic year, including reading speed and reading comprehension, and to examine the predictive value of baseline cognitive abilities for later reading performance in typically developing pre-primary school children.

**Methods:**

Fifty-four typically developing children (age = 75.41 ± 5.79 months; 57.4% male) enrolled in a local primary school participated in the study. Cognitive abilities, including attention, working memory, executive functions, and visual perception, were evaluated using standardized neuropsychological tests and parent-reported questionnaires. Reading performance (comprehension and speed) was assessed using a text and comprehension questions approved by the Ministry of National Education.

**Results:**

Spearman correlation analysis revealed statistically significant weak to moderate correlations between visuoperceptual skills (Words per minute (WPM): ρ = 0.335, *p* = 0.013; total text reading time (TRT): ρ = 0.315, *p* = 0.021), executive functions (TRT; ρ = 0.277, *p* = 0.042), and reading speed (WPM and TRT). In addition, reading comprehension was significantly correlated, with weak-to-moderate effect sizes, with working memory (ρ = 0.406, *p* = 0.002), executive functions (ρ = 0.272, *p* = 0.047), and visuoperceptual skills (ρ = 0.29, *p* = 0.033). Furthermore, suffix errors were moderately and significantly associated with visual discrimination skills (ρ = 0.473, *p*<0.001). Regression analyses indicated that working memory accounted for 23% of the variance in comprehension scores.

**Conclusion:**

These results suggest that working memory may be a key factor underlying reading comprehension. In this context, incorporating working memory training into early educational programs for typically developing pre-primary school children may enhance reading comprehension and indirectly contribute to academic success.

## 1 Introduction

Reading is a fundamental determinant of academic success, and reading skills acquired in early childhood have a decisive impact on learning outcomes in later years (Balansag, 2025). However, children show significant individual differences in the process of learning to read; some children complete this process relatively easily, while others experience difficulties from an early age (Ma et al., 2025). To support reading performance, identifying cognitive skills related to reading in the period before learning to read and integrating early interventions targeting these skills into educational settings is critically important (Welsh et al., 2010; Ne’eman and Shaul, 2021).

Reading has a complex, multidimensional, and dynamic structure (Safi et al., 2019). Effective reading performance requires an individual to be able to visually recognize words, match them with phonological, orthographic, and semantic representations stored in memory, and then integrate these representations with context to construct the meaning of the sentence and text. As indicated in the simple reading model, phonological processing involves the integration of visual attention and executive skills (Hoover and Gough, 1990; Taran et al., 2022). Similarly, working memory plays a crucial role in integrating vocabulary and syntactic knowledge (Daneman and Carpenter, 1980; Masson and Miller, 1983; Nouwens et al., 2021). Findings from neuroimaging studies also show that reading acquisition is based not only on linguistic processes but also on cognitive skills (Bailey et al., 2018; Habib, 2018). Furthermore, it is noted that in the early stages of reading acquisition, especially during the preschool period, reading is not yet automatic, so cognitive demands are higher, and as reading fluency is achieved, the use of cognitive resources changes (Logan, 1997; Schwanenflugel et al., 2006).

Numerous studies in the literature examine the relationship between cognitive skills and reading performance. For example, it has been reported that working memory explains a significant portion of the variance in literacy skills (Miller et al., 2013), that attention and inhibitory control skills are related to phonological processing (Nouwens et al., 2021; Öncü and Hiğde, 2025), and that visual skills show significant relationships with early reading accuracy and speed (Vernet et al., 2022). However, most of these studies have addressed cognitive skills individually or examined different cognitive domains in different samples. This makes it difficult to determine the relative contributions of cognitive skills supporting reading performance within the same developmental population. Furthermore, studies that evaluate non-linguistic cognitive processes, such as visual perception, together and simultaneously with other cognitive skills, such as attention and memory, are limited. However, it is known that reading performance is shaped by the interaction of multiple cognitive skills at different stages of the reading acquisition process, and the relative importance of these skills can vary depending on individual differences and task characteristics (Peng et al., 2018; Wu et al., 2020).

In this context, considering multiple cognitive skills together within the same sample in typically developing pre-primary school children with similar developmental characteristics is important in order to reveal which cognitive domains are more strongly associated with reading performance. Given the limited evidence regarding the relative contributions of multiple cognitive skills within the same developmental population, examining whether preschool cognitive abilities predict later reading performance may facilitate the early identification of children at risk for reading difficulties. Furthermore, identifying the cognitive skills that predict later reading outcomes may support the inclusion of targeted interventions aimed at strengthening these skills within preschool educational programs, thereby enhancing academic achievement in both typically developing children and those experiencing reading difficulties. Therefore, identifying the preschool cognitive abilities that predict subsequent reading performance is of considerable importance.

The aim of this study was to examine the prospective relationships between baseline cognitive skills, including working memory, executive function, attention, and visual perception, and later reading performance, including reading speed and reading comprehension, in typically developing pre-primary school children, and to identify the cognitive skills that most strongly predicted reading outcomes.

## 2 Materials and methods

### 2.1 Participants

This prospective correlational study was conducted between September and June 2023 at a local public school. A total of 65 children who were about to start primary school were initially recruited. The study included 54 children (Mean age = 75.41 months, SD = 5.79 months; male = 57.4%, female = 42.6%), after excluding those who did not meet the inclusion criteria (*n* = 11). Children aged 6 years and older, whose native language was Turkish and monolingual, who were starting primary school and had not yet acquired reading and writing skills were included in the study. Children with a diagnosis of neurological or psychiatric disorders, a diagnosis of dyslexia, or organic conditions such as visual impairment were excluded.

The sufficiency of the sample size was calculated using a power analysis conducted with the G*Power program (version 3.1). The minimum required sample size (total n) was determined to be 54 to achieve a statistical power of 0.80 at a significance level (α) of 0.05. Thus, the study was conducted with this sample size. All study procedures were performed in accordance with the principles of the Declaration of Helsinki. Written informed consent was obtained from the parents or legal guardians of all participants, and this study was approved by the XX Ethics Board/Committee (Approval No: E-10840098-772.02-8008).

### 2.2 Procedure

The study involved students from three different classes, all following a parallel instructional process. The teachers were informed about the study and their cooperation was obtained. For children whose parents approved participation, sociodemographic data were recorded, and baseline assessments were conducted at the beginning of the academic year, prior to the acquisition of reading skills. The Digit Span Forward and Cancelation Tests (Regular and Irregular Shapes) were administered to assess attention; the Digit Span Backward test was administered to assess working memory; the Head-Toes-Knees-Shoulders Test (HTKS) was administered to assess executive skills; Motor-Free Visual Perception Test-3 (MVPT-3) was administered to assess visual perception. All assessments were conducted face-to-face. Additionally, parents were also asked to complete The Executive Functions and Occupational Routines Scale (EFORTS) to provide parent-reported measures of executive functions.

Reading performance was assessed at the end of the academic year, after children had acquired foundational reading skills. Children who had learned to read were evaluated using a standardized reading text approved by the Ministry of National Education. The prospective relationships between baseline cognitive skills and subsequent reading outcomes, including reading speed and reading comprehension, were then examined statistically.

### 2.3 Measures

#### 2.3.1 Sociodemographic data form

The child’s age, gender and medical condition were recorded.

#### 2.3.2 Cancelation tests (CT)

This is a neuropsychological test developed by Weintraub and Mesulam (1987). The CT, included in the BILNOT Battery, has been standardized for both adults and children (Kiliç et al., 2002; Kurt et al., 2011). Studies conducted in our country have shown that CT scores are grouped under the factors of visual-spatial scanning, impulsivity, and reaction speed related to stimulus context (Kurt, 1998; Kurt et al., 2011). In our study, the CT was used to measure sustained attention, which falls under the scope of supervisory attention (Weintraub and Mesulam, 1987; Lezak et al., 2012).

#### 2.3.3 Digit span forward and backward

The Digit Span Subtest, a subtest of the Wechsler Intelligence Scale for Children, was used to assess attention and working memory (Wechsler, 1991). The Digit Span Forward task, which assesses attention, requires the child to repeat a two-digit number presented by the examiner. When the same digit is repeated correctly twice, the number of digits is increased until an error is made. When two errors are made, the test is terminated, and the Digit Span Forward score is determined. Digit Span Backward which assesses working memory, is similar to Digit Span Forward, with the only difference being that the child is asked to repeat the given numbers backward (Uluç et al., 2011).

#### 2.3.4 Head-Toes-Knees-Shoulders Test (HTKS)

This test measures children’s ability to regulate their behavior. Children are asked to do the opposite of what they are told. The task increases in complexity by including four body parts (head, toe, knee, and shoulder). Responses are scored from 0 to 2 (Cameron Ponitz et al., 2008; McClelland et al., 2014).

#### 2.3.5 The Executive Functions and Occupational Routines Scale (EFORTS)

This test assesses mental characteristics such as impulse control, verbal and nonverbal memory, self-regulation, problem-solving, and planning in children aged 6–12. It is scored by the family on a 1–5 Likert scale. It has three subsections covering morning-evening routines, play-fun, and social routines, and contains a total of 30 questions. Scored from 1 (never) to 5 (always), with higher scores indicating better executive control (Akyurek et al., 2023).

#### 2.3.6 Motor-Free Visual Perception Test-3(MVPT-3)

MVPT-3 consists of 9 subscales and 65 shapes: visual discrimination (1–8), shape formation (9–13), visual memory-I (14–21), visual proximity-I (22–34), visual discrimination (35–45), position in space (46–50), figure-ground (51–55), visual proximity-II (56–60), and visual memory-II (61–65). A total visual perception score is calculated (Harber, 1979; Colarusso and Hammill, 2003; Atasavun Uysal, 2009; Metin and Aral, 2016).

#### 2.3.7 Reading performance

Reading performance was assessed in terms of reading speed and reading comprehension. For the assessment, one of the texts provided by the Turkish Republic Ministry of National Education’s database was selected. The difficulty levels of these texts were determined by experts according to the children’s grade levels. The text used in the present study was randomly selected from among the texts approved as appropriate for first-grade students and consisted of 112 words.

*Reading comprehension* was assessed by asking the children to read the text aloud and answer comprehension questions. Assessments were administered individually by four trained examiners. Participants were randomly assigned to the examiners. To ensure procedural consistency, all examiners followed a standardized assessment protocol. Due to feasibility constraints and to minimize fatigue in young children, inter-rater reliability analysis was not performed. During oral reading, word-level errors, suffix errors, and spontaneous self-corrections were recorded in real time by the examiner on a printed copy of the text. The total number of errors was calculated based on these records. Reading comprehension was evaluated using seven open-ended questions based on a reading passage drawn from assessment materials officially developed and standardized by the Turkish Ministry of National Education. Six questions assessed factual information, and one question assessed inferential comprehension. Children provided oral responses to each question (e.g., answering “ladybug” to the question “Which kite did Karamel choose?”). Since most of the questions were factual in nature, responses could be readily scored as correct or incorrect. In cases where responses to the inferential question were ambiguous, scoring decisions were reviewed by the research team and finalized through consensus. Each correct response was awarded 1 point, yielding a maximum possible score of 7.

*Reading speed* was evaluated by recording the total time required to read the text aloud and calculating the number of words read per minute.

## 3 Statistical analysis

Statistical analyses were performed using IBM SPSS Statistics version 24. Descriptive statistics were used to summarize the demographic characteristics of the participants. Mean, standard deviation, and range were calculated for the continuous variable (age), while frequencies and percentages were used for the categorical variable (gender). The relationships between cognitive skills and reading performance were examined using Spearman’s rank correlation coefficient (ρ) as the data did not meet the assumptions of normality. Multiple linear regression analyses were conducted to examine whether cognitive variables predicted reading performance. Separate models were established for reading speed (WPM), total reading time (TRT), and reading comprehension (NCR). Age and gender were included as control variables in the models. Regression coefficients (B), standard errors (SE), standardized coefficients (β), *t*-values, and *p*-values were reported. A significance level of *p* < 0.05 was adopted for all statistical analyses. The strength of the correlations was interpreted according to the criteria proposed by Cohen (1988): correlation coefficients ρ < 0.30 were considered weak, 0.30 ≤ ρ < 0.50 moderate, 0.50 ≤ ρ < 0.70 strong, and ρ ≥ 0.70 very strong (Cohen, 1988).

## 4 Results

### 4.1 Demographic characteristics

All students’ demographic characteristics are presented in Table 1. The mean age of the children included in the study was 75.41 ± 5.79 months, and the proportion of boys and girls was 57.4 and 42.6%, respectively. Individual socioeconomic status (SES) analyses could not be performed due to missing data. However, according to the 2023 SES data published by Turkish Statistical Institute, the SES score of the district in which the study was conducted was reported as 151. This finding suggests that the study sample was located in a relatively advantaged, upper-middle socioeconomic environment (Turkish Statistical Institute (TurkStat), 2025). *Sosyoekonomik seviye, 2023*. TÜÝK Veri Portali.^1^

**TABLE 1:** Demographic characteristics of participants.

*N* = 54	Mean (SD)	Range
Age (month)	75.41 (5.79)	65–93
**Gender**	**n**	**%**
Male	31	57.4
Female	23	42.6

SD, standard deviation.

### 4.2 Cognitive and reading skills

The cognitive skills of the children were evaluated in four different domains at the beginning and at the end of the academic year. The children’s cognitive data are presented in Table 2. As cut-off values for these cognitive measures have not been established, they were not included in the table. However, all participants were confirmed to demonstrate typical development and had no clinical diagnoses.

**TABLE 2:** Descriptive statistics of cognitive data at the beginning of academic year.

Cognitive domains *N* = 54	Measurements	Mean	SD	Minimum	Maximum
W.M	DSBS	2,00	1,099	0	5
Executive functions	HTKS-TT	141.61	24.57	86	200
	HTKS-CR	29.78	8.41	6	40
	HTKS-TS	64.07	16.68	14	80
	EFORTS-MER	4.04	0.573	2.75	5
	EFORTS-PF	4.14	0.64	1.44	5
	EFORTS-SR	4.05	0.48	2.57	5
	EFORTS-TS	4.0804	0.48664	2.63	5
Visual perception	MVPT-VD	6.19	1.50	2	8
	MVPT-VFC	3.35	1.16	1	5
	MVPT-VM	4.33	1.87	1	8
	MVPT-SR	6.06	2.22	1	11
	MVPT-FG	3.19	1.17	1	5
	MVPTT-TS	23.06	4.87	10	36
Atten.	DSFS	5.81	1.183	3	8
	RCCR	32.25	8.99	13.00	52.00
	ICCR	42.48	9.83	20.00	58.00

SD, standard deviation; WM, working memory; Atten, attention; DSBS, Digit Span Backword Score; HTKS-TT, Head-Toes-Knees-Shoulders Test (HTKS) Total Time; HTKS-CR, HTKS Correct Responses; HTKS-TS, HTKS Total Score; EFORTS-MER, The Executive Functions and Occupational Routines Scale (EFORTS)– Morning–Evening Routines; EFORTS-PF, EFORTS–Play–Fun; EFORTS-SR, EFORTS–Social Routines; EFORTS-TS, EFORTS Total Score; MVPT-VD, Motor-Free Visual Perception Test (MVPT)–Visual Discrimination; MVPT-VFC, MVPT–Visual Form Constancy; MVPT-VM, MVPT–Visual Memory; MVPT-SR, MVPT–Spatial Relations; MVPT-FG, MVPT–Figure–Ground; MVPT-TS, MVPT Total Score; RCCR, Regular Cancelation Test Correct Responses; ICCR, Irregular Cancelation Test Correct Responses; DSFS, Digit Span Forward Score.

The children’s reading skills—including words read per minute, suffix errors, reading time, and number of correct answers—are presented in Table 3.

**TABLE 3:** Descriptive statistics of reading skills.

*N* = 54	Mean	SD	Minimum	Maximum
WPM	39.67	16.137	7	106
SE	0.72	1.054	0	4
TRT	181.76	55.52	63	308
NCR	3.44	1.690	1	7
CR (%)	49.22	24.13	14.00	100.00

SD, standard deviation; WPM, words per minute; SE, suffix errors; TRT, text reading time; NCR, number of correct responses; PCR, percentage of correct responses.

The relationship between the cognitive and reading skills was reported in Table 4. A moderate positive correlation was found between the digit span backward score and the number of correct answers (ρ = 0.406, *p* = 0.002). Similarly, a weak positive correlation was found between the number of correct answers and the total HTKS score (ρ = 0.272, *p* = 0.047) and the total MVPT score (ρ = 0.290, *p* = 0.033). The number of correct answers, representing reading comprehension, was found to be related to working memory, executive function, and visual perceptual skills.

**TABLE 4:** The relationship between the cognitive and reading skills.

Cognitive domains *N* = 54	Variable	WPM		SE		TRT		NCR	
	Measurements	Rho	*p*	Rho	*p*	Rho	*p*	Rho	*p*
WM	DSBS	0.247	0.072	0.081	0.558	–0.101	0.467	0.406	0.002**
Executive function skills	HKTS-TT	–0.148	0.285	–0.167	0.228	0.065	0.641	–0.088	0.529
	HKTS-TS	0.187	0.175	0.069	0.621	–0.079	0.568	0.272	**0.047** *
	HKTS-CR	0.238	0.084	0.11	0.429	–0.112	0.421	0.25	0.068
	EFORTS-MER	0.165	0.233	–0.038	0.786	–0.266	0.052	–0.078	0.577
	EFORTS-PF	0.007	0.961	–0.083	0.553	–0.086	0.535	–0.073	0.602
	EFORTS-SR	0.18	0.192	–0.088	0.528	–0.277	**0.042**	0.103	0.461
	EFORTS-TS	0.132	0.34	–0.078	0.575	0.001	0.996	–0.08	0.565
Visual perceptual skills	MVPT-VD	0.138	0.318	0.473	< 0.001**	–0.073	0.601	0.182	0.188
	MVPT-VFC	0.136	0.325	0.25	0.069	–0.148	0.286	0.244	0.075
	MVPT-VM	0.195	0.158	0.01	0.945	–0.277	**0.042** *	0.107	0.44
	MVPT-SR	0.252	0.066	–0.009	0.946	–0.305	**0.025** *	0.119	0.393
	MVPT-FG	0.265	0.055	0.241	0.082	–0.045	0.75	0.265	0.055
	MVPT-TS	0.335	**0.013** *	0.272	**0.047** *	–0.315	**0.021** *	0.290	**0.033** *
Atten. skills	DSFS	–0.125	0.368	–0.178	0.197	0.162	0.243	0.221	0.108
	RCCR	0.081	0.561	–0.038	0.785	–0.126	0.362	–0.108	0.436
	ICCR	0.062	0.654	0.057	0.683	–0.087	0.531	0.053	0.705

WPM, words per minute; SE, suffix errors; TRT, text reading time; NCR, number of correct responses; WM, working memory; Atten, Attention; DSBS, Digit Span Backward Score; HTKS-TT, Head-Toes-Knees-Shoulders Test (HTKS) Total Time; HTKS-CR = HTKS Correct Responses; HTKS-TS, HTKS Total Score; EFORTS-MER, The Executive Functions and Occupational Routines Scale (EFORTS)– Morning–Evening Routines; EFORTS-PF, EFORTS – Play–Fun; EFORTS-SR, EFORTS – Social Routines; EFORTS-TS, EFORTS Total Score; MVPT-VD, Motor-Free Visual Perception Test (MVPT)–Visual Discrimination; MVPT-VFC, MVPT – Visual Form Constancy; MVPT-VM, MVPT – Visual Memory; MVPT-SR, MVPT–Spatial Relations; MVPT-FG, MVPT – Figure–Ground; MVPT-TS, MVPT Total Score; RCCR, Regular Cancelation Test Correct Responses; ICCR, Irregular Cancelation Test Correct Responses; DSFS, Digit Span Forward Score.

*p < 0.05; **p < 0.01.

A moderate positive correlation was detected between words read per minute and the total MVPT score (ρ = 0.335, *p* = 0.013). A moderate negative correlation was found between total reading time and the total MVPT score (ρ = –0.315, *p* = 0.021) and MVPT-3 subarea of spatial relations (ρ = –0.305, *p* = 0.025, while a weak negative correlation was found between text reading time and the MVPT sub-area of visual memory (ρ = –0.277, *p* = 0.042). A weak negative correlation was also found between total reading time and the social routines sub-area of the EFORTS (ρ = –0.277, *p* = 0.042). It was noted that reading speed was particularly related to visual perceptual skills.

A moderate positive and statistically significant correlation was found between suffix errors and the MVPT sub-area of visual discrimination (ρ = 0.473, *p* = 0.000). A weak positive correlation was also found between the total MVPT score and suffix error (ρ = 0.272, *p* = 0.047). Visual perceptual skills were found to be related to suffix errors.

No statistically significant relationship was found between attention measures and reading skills.

In this study, a partial correlation analysis was conducted to examine whether the relationship between reading performance and cognitive skills remains significant after controlling for age and gender. This approach allowed us to assess the unique association between the variables while statistically accounting for potential confounding effects. Thus, the presence of a controlled relationship between the variables was evaluated prior to conducting the regression analysis. The results of this analysis are presented in Table 5. According to these findings, words per minute, an indicator of reading speed, demonstrated a moderate positive correlation with the MVPT total score (*r* = 0.307, *p* = 0.027). However, text reading time showed a weak but statistically significant negative correlation with the MVPT total score (*r* = –0.294, *p* = 0.034). A weak positive correlation was identified between the number of correct responses, reflecting reading comprehension, and the Digit Span Backward score (*r* = 0.432, *p* = 0.001). Additionally, a moderate positive correlation was observed between the number of correct responses and HTKS (*r* = 0.297, *p* = 0.033).

**TABLE 5:** Results of partial correlation.

Control variables (age and gender)		NCR	TRT	WPM
WM	*r*	0.432	–0.107	0.257
	*p*	0.001**	0.449	0.066
HKTS-TS	*r*	0.297	–0.093	0.208
	*p*	0.033*	0.514	0.139
EFORTS-SR	*r*	–0.078	–0.077	–0.022
	*p*	0.581	0,589	0.875
MVPT-VD	*r*	0.124	–0.033	0.092
	*p*	0.380	0.817	0.516
MVPT-VM	*r*	0.096	–0.277	0.194
	*p*	0.499	0.047*	0.168
MVPT-SR	*r*	0.093	–0.293	0.236
	*p*	0.514	0.035*	0.091
MVPT-TS	*r*	0.253	–0.294	0.307
	*p*	0.070	0.034*	0.027*

NCR, number of correct responses; TRT, text reading time; WPM, words per minute; WM, working memory; DSBS, Digit Span Backward Score; HTKS-TS, Head-Toes-Knees-Shoulders Test Total Score; EFORTS-SR, The Executive Functions and Occupational Routines Scale (EFORTS)– Social Routin; MVPT-VD, Motor-Free Visual Perception Test (MVPT)–Visual Discrimination; MVPT-VM, MVPT– Visual Memory; MVPT-SR, Motor-Free Visual Perception Test–Spatial Relations; MVPT-TS, MVPT Total Score.

*p < 0.05; **p < 0.01.

### 4.3 Results of generalized linear regression analyses

After partial correlation analysis, a generalized linear regression model was applied to examine the predictors in more detail by controlling for age and gender variables. Three different models, in which all cognitive skills were entered simultaneously, were tested for reading performance parameters.

*In Model 1*, all cognitive skills, age, and gender were entered as predictors of NCR. The model was found to be statistically significant, accounting for 23 % of the variance (*R*^2^ = 0.23, *F* = 2.87, *p* = 0.02), DSBS was identified as a significant predictor of NCR (*B* = 0.54, SE = 0.23, β = 0.35, *t* = 3.34, *p* = 0.023). The effects of age (*B* = 0.05, SE = 0.04, β = 0.17, *p* = 0.218) and gender (*B* = –0.57, SE = 0.47, β = –0.33, *p* = 0.23) were not found to be statistically significant (Table 6).

**TABLE 6:** Results of multiple linear regression analyses.

Model 1			NCR		
R2 = 0.23, Adj R2 = 0.15, F = 2.87, p (Model) = 0.02, AIC/BIC = 209/223					
**Predictors**	***B***	***SE***	**β**	***t***	***P***
DSBS	0.54	0.23	0.35	2.34	**0.023**
HTKS	0.00	0.01	0.08	0.59	0.557
MVPTT	0.03	0.05	0.08	0.61	0.542
Age	0.05	0.04	0.17	1.24	0.218
Gender	–0.57	0.47	–0.33	-1.20	0.235
**Model 2**			**WPM**		
R2 = 0.15, Adj R2 = 0.016, F = 1.73, p (Model) = 0.147, AIC/BIC = 458/472					
**Predictors**	***B***	***SE***	**β**	***t***	***P***
DSBS	2.14	2.32	0.14	0.925	0.359
HTKS	0.04	0.15	0.04	0.297	0.768
MVPTT	0.79	0.50	0.23	1.559	0.125
Age	0.41	0.41	0.14	1.014	0.316
Gender	-1.02	4.76	–0.06	–0.215	0.830
**Model 3**			**TRT**		
R2 = 0.10, Adj R2 = 0.01, F = 1.12, p (Model) = 0.363, AIC/BIC = 594/608					
**Predictors**	***B***	***SE***	**β**	***t***	***P***
DSBS	–0.42	8.20	–0.00	–0.05	0.959
HTKST	0.08	0.54	0.024	0.15	0.880
MVPTT	–3.53	1.79	–0.30	-1.96	0.055
Age	–0.60	1.45	–0.06	–0.41	0.677
Gender	–0.42	8.20	–0.00	–0.08	0.935

NCR, number of correct responses; WPM, words per minute; TRT, text reading time; B, regression coefficient; SE, standard error; β, standardized regression coefficient; DSBS, Digit Span Backward Score; HTKS, Head-Toes-Knees-Shoulders Test; MVPT, Motor-Free Visual Perception Test; *p*, *p*-value; Bold*: *p* < 0.05.

To further evaluate the contributions of visual perception (MVPT) and executive functions (HTKS), three additional models were tested for reading comprehension performance. However, in these models, neither HTKS nor MVPT scores emerged as significant predictors, and the explanatory power of the models did not increase substantially. The consistent significance of the models including DSBS, together with the significant contribution of DSBS within these models, suggests that DSBS is a meaningful predictor of reading comprehension performance. These additional analyses are presented as Supplementary material.

*In Model 2*, all cognitive skills, age, and gender were entered as predictors of WPM. The model was not statistically significant (*R*^2^ = 0.15, *F* = 1.73, *p* = 0.147). None of the cognitive variables significant predictors of WPM, including DSBS (B = 2.14, SE = 2.32, β = 0.14, *p* = 0.359), HTKS (B = 0.04, SE = 0.15, β = 0.04, *p* = 0.768), and MVPTT (*B* = 0.79, SE = 0.50, β = 0.23, *p* = 0.125). Similarly, age (*B* = 0.41, SE = 0.41, β = 0.14, *p* = 0.316) and gender (*B* = -1.02, SE = 4.76, β = –0.06, *p* = 0.830) were not significant predictors (Table 6).

*In Model 3*, all cognitive skills, age, and gender were entered as predictors of TRT. The model was not statistically significant (*R*^2^ = 0.10, F = 1.12, *p* = 0.363). None of the predictors reached statistical significance. Although MVPTT showed a trend toward significance (*B* = –3.53, SE = 1.79, β = –0.30, *p* = 0.055), this association did not reach conventional levels of statistical significance. The effects of DSBS (B = –0.42, SE = 8.20, β = –0.00, *p* = 0.959), HTKS (B = 0.08, SE = 0.54, β = 0.024, *p* = 0.880), age (*B* = –0.60, SE = 1.45, β = –0.06, *p* = 0.677), and gender (*B* = –0.42, SE = 8.20, β = –0.00, *p* = 0.935) were also not significant (Table 6).

## 5 Discussion

The present study examined the prospective relationship between cognitive abilities assessed at the beginning of the academic year and reading skills acquired by the end of the academic year, as well as the predictive value of these baseline cognitive abilities for later reading performance in typically developing pre-primary school children. The findings can be summarized within two main domains: (1) the relationship between reading speed, visual perception, and executive functions, and (2) the relationship between reading comprehension, visual perception, working memory, and executive functions. Furthermore, working memory was found to significantly predict reading comprehension, accounting for approximately 23% of the variance. In terms of reading speed, a weak to moderate correlation was observed between reading speed and visual perception and executive functions. Although visual perceptual skills accounted for approximately 10% of the variance in total reading time, this association was only marginally significant, and the overall regression model did not reach statistical significance. Therefore, these variance ratios should be interpreted cautiously. Although the regression model was not statistically significant, the observed correlation between visual perceptual skills and reading comprehension (NCR) warrants further discussion. Herein, it should be noted that our present findings are consistent with theoretical explanations that conceptualize reading as a visually challenging cognitive process. Neuroimaging studies on reading have revealed that visual processing areas such as the occipitotemporal and temporoparietal areas show increased activation during reading (Sarkari et al., 2002; Landi et al., 2013). Indeed, various visual perceptual skills including figure-ground perception, visual closure, visuospatial perception, and spatial position perception are necessary for distinguishing letters and words from surrounding stimuli and for identifying letters across varying spatial positions during reading acquisition (Woodrome and Johnson, 2009; Lipowska et al., 2011; Baluoti et al., 2012; Bellocchi et al., 2017). Studies in preschool children have shown that visual perception skills are related to reading ability and play a significant role in naming speed (Ammawat et al., 2019; Saneyoshi and Inada, 2025). In this context, the association identified between early reading speed and visual perception abilities in children assessed immediately prior to formal schooling extends previous findings in the literature.

In terms of reading comprehension, we observed weak to moderate correlations with visual perception, working memory, and executive functions. These associations remained significant for working memory and executive functions after conducting partial correlation analyses. Our regression analysis also suggested a relationship between working memory functions and reading comprehension, indicating the main predictive role of working memory in reading comprehension. Here, it is important to consider the existing literature on the role of working memory in reading comprehension. Daneman and Carpenter suggested that reading comprehension is a complex cognitive process that requires the storage, analysis, and integration of pragmatic, semantic, and syntactic information. In that context, working memory, which is assumed to involve both processing and storage functions, may influence reading comprehension both directly and indirectly (Baddeley and Hitch, 1974; Daneman and Carpenter, 1980; Nouwens et al., 2021). Indeed, previous studies have suggested that working memory has a strong correlation with reading comprehension and can predict reading comprehension (Butterfuss and Kendeou, 2018; Follmer, 2018; Wu et al., 2020). Consistent with this literature, our study revealed that working memory predicted 23% of the variance in the number of correct answers reflecting reading comprehension performance. This finding should also be interpreted in light of the specific characteristics of the comprehension task used in the present study. The questions predominantly consisted of factual questions (6 out of 7 items), with only one inferential question. Therefore, performance likely relied primarily on the encoding, temporary maintenance, and retrieval of explicitly stated textual information rather than on higher-order inferential integration. This task structure may therefore have increased the relative contribution of working memory to performance (Daneman and Carpenter, 1980; Butterfuss and Kendeou, 2018). Beyond working memory, it has been suggested that executive functions, which have many sub-components, are also associated with reading comprehension in early literacy development (Christopher et al., 2012; Jacob and Parkinson, 2015; Follmer, 2018; Cirino et al., 2019; Nouwens et al., 2021). For the development of comprehension skills during reading, inhibiting irrelevant information may be necessary in order to integrate new relevant information into memory and/or existing background knowledge (Wu et al., 2020). In line with this view, Kieffer et al. (2013) reported that response inhibition, a core component of executive functions, significantly contributes to reading comprehension. Similarly, a recent meta-analysis demonstrated a significant but weak association between dominant response inhibition and reading comprehension (Kieffer et al., 2013; Follmer, 2018; Nouwens et al., 2021). Consistent with these findings, our present study found a weak positive association with the performance on the HTKS test (McClelland et al., 2014), which has already been shown to measure executive functions, particularly those assessing inhibitory control, and reading comprehension (McClelland et al., 2014).

From another perspective, it is important to consider that the morphological, orthographic, and phonological characteristics of a language may impose different cognitive demands and thereby influence reading performance. Logographic writing systems and orthographies with greater visual complexity may place stronger demands on visual processing skills, whereas morphologically complex languages may increase working memory load during reading (Tong and McBride-Chang, 2010; Yang et al., 2019). In this context, Turkish is an agglutinative language in which complex word forms are created through the sequential addition of morphemes, while also being characterized by complete orthographic transparency (Miller et al., 2019). The suffixing morphological structure of Turkish may increase working memory demands during word decoding and integration processes, which is consistent with our finding that working memory emerged as a significant predictor of reading comprehension. Furthermore, the fact that all participants were monolingual and had not yet begun learning a second language eliminates potential confounding effects associated with bilingualism (Tong and McBride-Chang, 2010), allowing the findings to be interpreted more clearly within the structural characteristics of Turkish.

The absence of a significant effect of attention on reading speed and comprehension warrants further discussion. In detail, although there was a relationship between working memory, executive function, and visual perception skills and reading performance, we observed no statistically significant relationship between attention skills and reading performance. In contrast to our findings, a recent neuroimaging study conducted on typically developing children found that while multiple brain regions are activated during reading, the highest level of activation occurred within attentional networks, particularly the frontoparietal network (Bailey et al., 2018). This finding is also consistent with previous work by McClelland indicating a significant relationship between attention and reading, suggesting that attention levels in early childhood are strong predictors of long-term academic achievement (McClelland et al., 2014). However, despite this evidence, certain factors such as attention type, age range, and sample characteristics might bias this relationship (Steele et al., 2012). In support of this, in the present study, we assessed only the attention subtypes of sustained attention and simple attention skills and found no significant relationship between these subtypes and reading speed and reading comprehension. Hence, it is not surprising to hypothesize that the following factors might be responsible for this discrepancy. First, the subtypes of attention we measured in our study such as sustained attention and simple attention skills, may not be related to reading speed and reading comprehension at this age. This is supported by Steele and colleagues, who reported that sustained and selective attention in children aged 3–6 years are associated with mathematics skills but not with reading skills (Steele et al., 2012). Second, the differences in the assessment tools observed between our study and previous studies may explain the discrepancy. This has also been suggested in previous literature that cognitive assessment tools have varying levels of sensitivity (Salthouse, 2010), and that might alter research outcomes (Lezak et al., 2012).

### 5.1 Limitations

Despite providing valuable data to the current literature, this study has several limitations that should be acknowledged. First, the relatively small sample size and the recruitment of participants from a single school limit the generalizability of the findings. Second, the gestational age of the children included in the study was not recorded, which may have influenced developmental variability. Third, executive functions were assessed using a measure that was partially based on parent reports, which may be susceptible to reporting bias. Fourth, socioeconomic status was not systematically analyzed due to incomplete demographic data, despite its potential influence on both working memory and reading-related outcomes. Finally, as multiple statistical comparisons were conducted without formal correction procedures, the possibility of Type I error should be considered when interpreting the findings. Future research with larger and more diverse samples is warranted to further elucidate the dynamic interaction between cognitive skills and reading ability.

Nevertheless, despite these limitations, this study has several strengths. First, the prospective nature of the study enabled the investigation of how early cognitive abilities were related to later reading performance. Furthermore, unlike much of the existing literature, which has focused primarily on children with reading difficulties or diagnosed disorders, this study was conducted with typically developing pre-primary school children—a population for which research is relatively limited. Moreover, visual perception, working memory, executive functions, and attention were simultaneously assessed within the same sample, providing a comprehensive view of cognitive contributions to reading. Finally, our findings highlighted working memory as a key predictor of reading comprehension, underscoring its central role in early literacy development.

## 6 Conclusion

To conclude, the results of this study support the idea that working memory plays a predictive role in reading comprehension skills. However, the effects of attention and visual perceptual skills on reading performance in pre-primary school children require further longitudinal studies.

## Data Availability

The raw data supporting the conclusions of this article will be made available by the authors, without undue reservation.

## Footnotes

1
https://veriportali.tuik.gov.tr/tr/press/57942/metadata

## References

[B1] Akyurek G. Efe A. Bumin G. (2023). Turkish adaptation of the executive functions and occupational routines scale: Validity and reliability among children with dyslexia. *Percept. Mot. Skills* 130 364–385. 10.1177/00315125221142650 36445859

[B2] Ammawat W. Attanak A. Kornpetpanee S. Wongupparaj P. (2019). Pre-schoolers’ visual perception and attention networks influencing naming speed: An individual difference perspective. *Heliyon* 5:e02587. 10.1016/j.heliyon.2019.e02587 31660445 PMC6806663

[B3] Atasavun Uysal S. (2009). *The Comparison of the Effect of Two Different Visual Perception Treatment in Low Vision Children. Hacettepe Üniversitesi / Sağlık Bilimleri Enstitüsü / İş ve Uğraşı Tedavisi Ana Bilim Dalı.* Available online at: https://tez.yok.gov.tr/UlusalTezMerkezi/tezDetay.jsp?id=OyKF5W3F0cljXMi82fWt0Q&no=ZB6U0iHQytDtRQOMVHJEvw (Accessed December 14, 2025).

[B4] Baddeley A. D. Hitch G. J. (1974). “Working memory,” in *Psychology of Learning and Motivation*, ed. Bower G. A. (New York, NY: Academic Press), 47–89. 10.1016/S0079-7421(08)60452-1

[B5] Bailey S. K. Aboud K. S. Nguyen T. Q. Cutting L. E. (2018). Applying a network framework to the neurobiology of reading and dyslexia. *J. Neurodev. Disord.* 10:37. 10.1186/s11689-018-9251-z 30541433 PMC6291929

[B6] Balansag J. P. (2025). Reading performance as a predictor of academic success of key stage 2 learners. *Int. J. Res. Innov. Soc. Sci.* IX, 1932–1949. 10.47772/IJRISS.2025.903SEDU0149

[B7] Baluoti A. Bayat M. Alimoradi M. (2012). Relationship between visual perception and reading disability in primary students of Ahwaz city. *Res. J. Appl. Basic Sci.* 10 2091–2096.

[B8] Bellocchi S. Muneaux M. Huau A. Lévêque Y. Jover M. Ducrot S. (2017). Exploring the link between visual perception, visual-motor integration, and reading in normal developing and impaired children using DTVP-2. *Dyslexia* 23 296–315. 10.1002/dys.1561 28691167

[B9] Butterfuss R. Kendeou P. (2018). The role of executive functions in reading comprehension. *Educ. Psychol. Rev.* 30 801–826. 10.1007/s10648-017-9422-6

[B10] Cameron Ponitz C. E. McClelland M. M. Jewkes A. M. Connor C. M. Farris C. L. Morrison F. J. (2008). Touch your toes! Developing a direct measure of behavioral regulation in early childhood. *Early Child. Res. Q.* 23 141–158. 10.1016/j.ecresq.2007.01.004

[B11] Christopher M. E. Miyake A. Keenan J. M. Pennington B. DeFries J. C. Wadsworth S. J. et al. (2012). Predicting word reading and comprehension with executive function and speed measures across development: A latent variable analysis. *J. Exp. Psychol. Gen.* 141 470–488. 10.1037/a0027375 22352396 PMC3360115

[B12] Cirino P. T. Miciak J. Ahmed Y. Barnes M. A. Taylor W. P. Gerst E. H. (2019). Executive function: Association with multiple reading skills. *Read. Writ.* 32 1819–1846. 10.1007/s11145-018-9923-9 31680727 PMC6824553

[B13] Cohen J. (1988). *Statistical Power Analysis for the Behavioral Sciences*, 2nd Edn. New York, NY: Routledge. 10.4324/9780203771587

[B14] Colarusso R. P. Hammill D. D. (2003). Motor-free visual perception test. *J. Psychoeduc. Assess.* 24 265–272. 10.1177/0734282906286339

[B15] Daneman M. Carpenter P. A. (1980). Individual differences in working memory and reading. *J. Verbal Learn. Verbal Behav.* 19 450–466. 10.1016/S0022-5371(80)90312-6

[B16] Follmer D. J. (2018). Executive function and reading comprehension: A meta-analytic review. *Educ. Psychol.* 53 42–60. 10.1080/00461520.2017.1309295

[B17] Habib S. (2018). *Learning and Teaching British Values.* Cham: Springer International Publishing. 10.1007/978-3-319-60381-0

[B18] Harber J. R. (1979). Perception and perceptual-motor integration: There is a difference. *Percept. Mot. Skills* 49 917–918. 10.2466/pms.1979.49.3.917 530792

[B19] Hoover W. A. Gough P. B. (1990). The simple view of reading. *Read. Writ.* 2 127–160. 10.1007/BF00401799

[B20] Jacob R. Parkinson J. (2015). The potential for school-based interventions that target executive function to improve academic achievement. *Rev. Educ. Res.* 85 512–552. 10.3102/0034654314561338

[B21] Kieffer M. J. Vukovic R. K. Berry D. (2013). Direct and indirect roles of executive functioning in reading comprehension for students in urban fourth grade classrooms. *Read. Res. Q.* 48 333–348. 10.1002/rrq.54

[B22] Kiliç B. G. Irak M. Koçkar A. I. Şener Ş. Karakaş S. (2002). Işaretleme testi türk formu’nun 6-11 yaş grubu çocuklarda standardizasyon çalişması^#^. *Klinik Psikiyatri Dergisi* 5 213–228.

[B23] Kurt M. (1998). *Sağ Serebral Hemisferin Bilişsel Işlevlerine Duyarlı Nöropsikolojik Testlerin Faktör Yapısının Incelenmesi. Hacettepe Üniversitesi / Sosyal Bilimler Enstitüsü / Psikoloji Ana Bilim Dalı.* Available online at: https://tez.yok.gov.tr/UlusalTezMerkezi/tezDetay.jsp?id=Sv5XOalXQXbhK6-BzPWueQ&no=Sv5XOalXQXbhK6-BzPWueQ (Accessed November 29, 2025).

[B24] Kurt P. Yener G. Oguz M. (2011). Impaired digit span can predict further cognitive decline in older people with subjective memory complaint: A preliminary result. *Aging Ment. Health* 15 364–369. 10.1080/13607863.2010.536133 21491221

[B25] Landi N. Frost S. J. Menc W. E. Sandak R. Pugh K. R. (2013). Neurobiological bases of reading comprehension: Insights from neuroimaging studies of word level and text level processing in skilled and impaired readers. *Read. Writ. Q.* 29 145–167. 10.1080/10573569.2013.758566 23662034 PMC3646421

[B26] Lezak M. D. Howieson D. B. Bigler E. D. Tranel D. (2012). *Neuropsychological Assessment*, 5th Edn. New York, NY: Oxford University Press.

[B27] Lipowska M. Czaplewska E. Wysocka A. (2011). Visuospatial deficits of dyslexic children. *Med. Sci. Monit.* 17 CR216–CR221. 10.12659/MSM.881718 21455108 PMC3539530

[B28] Logan G. D. (1997). Automaticity and reading: Perspectives from the instance theory of automatization. *Read. Writ. Q.* 13 123–146. 10.1080/1057356970130203

[B29] Ma L. Wang Y. Wang J. Chen R. Zhao G. Pan Z. et al. (2025). Reciprocal predictions between reading achievement and cognitive flexibility development in children and the mediating roles of the left middle frontal gyrus. *Hum. Brain Mapp.* 46:e70309. 10.1002/hbm.70309 40908812 PMC12411736

[B30] Masson M. E. J. Miller J. A. (1983). Working memory and individual differences in comprehension and memory of text. *J. Educ. Psychol.* 75 314–318. 10.1037/0022-0663.75.2.314

[B31] McClelland M. M. Cameron C. E. Duncan R. Bowles R. P. Acock A. C. Miao A. et al. (2014). Predictors of early growth in academic achievement: The head-toes-knees-shoulders task. *Front. Psychol.* 5:599. 10.3389/fpsyg.2014.00599 25071619 PMC4060410

[B32] Metin Ş Aral N. (2016). Proje yaklaşımına dayalı eğitimin beş yaş (60-72 ay) çocuklarının görsel algı becerilerine etkisinin belirlenmesi. *Eğitim Bilim* 41 149–162. 10.15390/EB.2016.4594

[B33] Miller M. Müller U. Giesbrecht G. Carpendale J. Kerns K. (2013). The contribution of executive function and social understanding to preschoolers’ letter and math skills. *Cogn. Dev.* 28 331–349. 10.1016/j.cogdev.2012.10.005

[B34] Miller P. Guldenoglu B. Kargin T. (2019). Reading failure in a completely transparent orthography representing a morphologically highly complex agglutinative language: The case of Turkish. *J. Dev. Phys. Disabil.* 31 669–689. 10.1007/s10882-019-09667-3

[B35] Ne’eman A. Shaul S. (2021). Readiness or impairment: Cognitive and linguistic differences between children who learn to read and those who exhibit difficulties with reading in kindergarten compared to their achievements at the end of first grade. *Front. Psychol.* 12:614996. 10.3389/fpsyg.2021.614996 33716879 PMC7947890

[B36] Nouwens S. Groen M. A. Kleemans T. Verhoeven L. (2021). How executive functions contribute to reading comprehension. *Br. J. Educ. Psychol.* 91:e12355. 10.1111/bjep.12355 32441782 PMC7983997

[B37] Öncü B. Hiğde A. Y. (2025). The predictive role of attention and listening comprehension in phonological awareness skills in 60- to 72-month-old preschoolers. *Int. J. Educ. Spect.* 7 116–130. 10.47806/ijesacademic.1604437

[B38] Peng P. Barnes M. Wang C. Wang W. Li S. Swanson H. L. et al. (2018). A meta-analysis on the relation between reading and working memory. *Psychol. Bull.* 144 48–76. 10.1037/bul0000124 29083201

[B39] Safi D. Lefebvre P. Nader M. (2019). Literacy acquisition: Reading development. *Handb. Clin. Neurol.* 173 185–199. 10.1016/B978-0-444-64150-2.00017-4 32958173

[B40] Salthouse T. A. (2010). *Major Issues in Cognitive Aging.* New York, NY: Oxford University Press.

[B41] Saneyoshi A. Inada N. (2025). Developmental relationships between visual cognitive skills and hiragana reading in Japanese children aged 4–8 years: Insights from mirror letter reading task. *Acta Psychol.* 260:105646. 10.1016/j.actpsy.2025.105646 41072294

[B42] Sarkari S. Simos P. G. Fletcher J. M. Castillo E. M. Breier J. I. Papanicolaou A. C. (2002). Contributions of magnetic source imaging to the understanding of dyslexia. *Semin. Pediatr. Neurol.* 9 229–238. 10.1053/spen.2002.35506 12350044

[B43] Schwanenflugel P. J. Meisinger E. B. Wisenbaker J. M. Kuhn M. R. Strauss G. P. Morris R. D. (2006). Becoming a fluent and automatic reader in the early elementary school years. *Read. Res. Q.* 41 496–522. 10.1598/RRQ.41.4.4 20072665 PMC2805254

[B44] Steele A. Karmiloff-Smith A. Cornish K. Scerif G. (2012). The multiple subfunctions of attention: Differential developmental gateways to literacy and numeracy. *Child Dev.* 83 2028–2041. 10.1111/j.1467-8624.2012.01809.x 22804751

[B45] Taran N. Farah R. DiFrancesco M. Altaye M. Vannest J. Holland S. et al. (2022). The role of visual attention in dyslexia: Behavioral and neurobiological evidence. *Hum. Brain Mapp.* 43 1720–1737. 10.1002/hbm.25753 34981603 PMC8886655

[B46] Tong X. McBride-Chang C. (2010). Chinese-English biscriptal reading: Cognitive component skills across orthographies. *Read. Writ.* 23 293–310. 10.1007/s11145-009-9211-9

[B47] Turkish Statistical Institute (TurkStat) (2025). *Socioeconomic Level, 2023.* Ankara: TurkStat Data Portal.

[B48] Uluç S. Öktem F. Erden H. G. Gençöz T. Sezgin N. (2011). Wechler çocuklar için zeka ölçeği-IV: Klinik bağlamda zekanın değerlendirilmesinde Türkiye için yeni bir dönem. *Türk Psikoloji Yazıları* 14 49–59.

[B49] Vernet M. Bellocchi S. Leibnitz L. Chaix Y. Ducrot S. (2022). Predicting future poor readers from pre-reading visual skills: A longitudinal study. *Appl. Neuropsychol. Child* 11 480–494. 10.1080/21622965.2021.1895790 33730530

[B50] Wechsler D. (1991). *Wechsler Intelligence Scale for Children (WISC-III).* Available online at: https://www.scirp.org/reference/referencespapers?referenceid=1268689 (Accessed December 3, 2025).

[B51] Weintraub S. Mesulam M. M. (1987). Right cerebral dominance in spatial attention. Further evidence based on ipsilateral neglect. *Arch. Neurol.* 44 621–625. 10.1001/archneur.1987.00520180043014 3579679

[B52] Welsh J. A. Nix R. L. Blair C. Bierman K. L. Nelson K. E. (2010). The development of cognitive skills and gains in academic school readiness for children from low-income families. *J. Educ. Psychol.* 102 43–53. 10.1037/a0016738 20411025 PMC2856933

[B53] Woodrome S. E. Johnson K. E. (2009). The role of visual discrimination in the learning-to-read process. *Read. Writ.* 22 117–131. 10.1007/s11145-007-9104-8

[B54] Wu Y. Barquero L. A. Pickren S. E. Taboada Barber A. Cutting L. E. (2020). The relationship between cognitive skills and reading comprehension of narrative and expository texts: A longitudinal study from Grade 1 to Grade 4. *Learn. Individ. Differ.* 80:101848. 10.1016/j.lindif.2020.101848 32536780 PMC7291864

[B55] Yang X. Peng P. Meng X. (2019). Contributions of basic cognitive processing to Chinese reading: The mediation effect of basic language processing. *Front. Psychol.* 9:2670. 10.3389/fpsyg.2018.02670 30671004 PMC6331404

