# Supplementary material for: The relationship between cognitive and reading skills in typically developing pre-primary school children
Source: Front Behav Neurosci. 2026 Jul 1;20:1832008. doi: 10.3389/fnbeh.2026.1832008 (PMC13371315; doi:10.3389/fnbeh.2026.1832008)
Supplement: Supplementary file 1 [file Data_Sheet_1.PDF]

Supplementary Material

Additional Anaylsis for Working Memory

**MODEL 1**			***NCR***		
R^2 = 0 .23 Adj R^2 = 0 .15 F = 2.87 P(Model) = ***0.02*** *AIC/BIC=209/223*					
Predictors	*B*	*SE*	*β*	t	*P*
DSBS	0.54	0.23	0.35	2.34	**0.023**
HSKT	0.00	0.01	0.08	0.59	0.557
MVPTT	0.03	0.05	0.08	0.61	0.542
Age	0.05	0.04	0.17	1.248	0.235
Gender	-0.57	0.47	-0.33	-1.203	0.218

**MODEL 2**			***NCR***		
R^2 =0.22 Adj R^2 = 0.16 F = 3.54 P(Model) = 0.013 AIC/BIC=207/219					
Predictors	*B*	*SE*	*β*	t	*P*
DSBS	0.57	0.22	0.37	-0.832	**0.013**
HSKT	0.01	0.01	0.11	0.76	0.446
Age	0.05	0.03	0.20	1.50	0.139
Gender	-0.65	0.45	-0.38	-1.44	0.156

**MODEL 3**			***NCR***		
R^2 =0.22 Adj R^2 = 0.16 F = 3.55 P(Model) = 0.013 AIC/BIC=207/219					
Predictors	*B*	*SE*	β	t	*P*
DSBS	0.59	0.21	0.38	2.8	**0.006**
MVPTT	0.03	0.04	0.11	0.78	0.435
Age	0.04	0.04	1.18	0.24	0.163
Gender	-0.54	0.47	-1.16	0.24	-0.325

**MODEL 4**			***NCR***		
R^2 =0.21 Adj R^2 = 0.17 F = 4.56 P(Model) = 0.007 AIC/BIC=206/216					
Predictors	*B*	*SE*	*β*	t	*P*
DSBS	0.65	0.19	0.42	**3.38**	**0.001***
Age	0.05	0.03	0.19	1.45	0.153
Gender	-0.64	0.45	-0.19	-1.44	0.156

NCR=Number of Correct Responses; DSBS= Digit Spa n Backward Score; HTKS= Heal Toes Knee Score; MVPT: Motor-Free Visual Perception Test * p < .05; ** p <.01
